# Integrated Physical Modeling and Optimal Control Method of Limited-Angle Torque Motor in Fuel Metering Apparatus

**DOI:** 10.3390/mi13060949

**Published:** 2022-06-15

**Authors:** Qian Chen, Hanlin Sheng, Shengbin Jiang

**Affiliations:** 1 College of Energy and Power Engineering, Nanjing University of Aeronautics and Astronautics, Nanjing 210016, China; freeflycq@nuaa.edu.cn; 2 Shenyang Institute of Automation, Chinese Academy of Sciences, Shenyang 110117, China; lifejsb@foxmail.com

**Keywords:** limited-angle torque motor, cascade PID control, PSO, physical modeling, control parameter tuning

## Abstract

Limited-angle torque motor (LATM) is a critical component to precisely drive the valve angle of an engine’s fuel metering apparatus and accurately measure the fuel flow, and research on it is of great significance. Thus, the LATM of a certain kind is regarded as the research object in this paper. Firstly, a Simscape-based LATM integrated physical modeling method is proposed, which can better demonstrate the real operational characteristics of a motor, compared with the current mathematical model. Secondly, a Proportional-Integral-Derivative (PID) parameter self-tuning method based on a constriction factor particle swarm optimization (CPSO) algorithm is broached since it is difficult to tune due to a large number of multi-loop cascade PID control parameters. Simulation and experimental results showed that the control performance increases by 40% in the triple closed-loop PID control system with a stronger disturbance rejection, simpler design, and quickly responds when compared with the previous empirical tuning method. The triple closed-loop PID control system comprises an angle loop + angle velocity loop + current loop and technically supports the engineering application design of motors.

## 1. Introduction

In aviation, whether fuel metering apparatus can precisely control the fuel flow is a prerequisite for an engine or an aircraft to operate safely and reliably. The key to realizing the “precise control” lies in whether a limited-angle torque motor (LATM) regulating the valve angle can perform precise control [1,2]. Hence, it is essential and crucial to research the LATM of fuel metering apparatus.

An LATM is characterized by small size, fast dynamic response, high steady-state accuracy, and high environmental adaptability [3,4,5]. Thus, many researchers have studied it carefully on LATMs. For example, Tsai et al. [6] designed a two-pole brushless DC LATM with a toroidally wound armature using selected ferromagnetic material and rare-earth permanent magnets. A designed position controller of LATM is applied to the fuel control of gas turbine engine, and experiments verified the effectiveness and practicability of the motor. Subsequently, using magnetic equivalent circuit modeling, Nasiri-Zarandi et al. [7] presented a two-pole brushless DC LATM with a toroidally wound armature. They introduced a new segmented rotor pole tip structure of the LATM, and the experimental results demonstrated its correctness. Wu et al. [8] proposed a toroidally wound radial-flux Halbach array permanent magnet LATM. Based on this type of motor, a fully parameterized and flexible equivalent magnetic circuit model arranged in matrix form employing Kirchhoff’s current laws is also proposed to improve the computational efficiency and extensibility. Meanwhile, a multi-objective optimization process is used to obtain the Pareto front of the desired objectives. Hekmati et al. [9] analyzed the outer rotor structure of slotless radial flux permanent magnet limited-angle torque motor with disturbed armature winding. The exact 2-D analytical modeling is applied to obtain the magnetic field parameters of the windings and excitation for both the outer rotor and the conventional inner rotor topologies. Experimental results showed good accuracy of the theoretical design and numerical simulations. The comparison results showed that the performance of the new actuator with outer rotor structure is significantly better. Li et al. [10] conducted a study on a novel LATM with an irregular slot number and established the relationship between its performance and main parameters according to analytical equations that are derived for predicting the torque-angle characteristic of the LATM, thus providing theoretical support for LATMs’ optimal design.

In recent years, researchers have also focused on LATM modeling and control. For example, in order to realize accurate tracking control of LATM with 50 Hz sawtooth reference waveform as input, Zhang et al. [11] established a dual closed-loop linear controller based on angle loop + current loop according to a nonlinear motor mathematical model and designed a gain scheduling controller for linear dynamic compensation, which effectively compensates the nonlinear variation of motor parameters. However, it has a poor control effect in the case of adding a load. Xiao et al. [12] built up an LATM position servo system based on a triple closed-loop PID control strategy comprised of angle loop + velocity loop + current loop, and successfully applied it in practical production, but no study on parameter self-tuning was conducted, and parameters were tuned completely based on experience. Li et al. [13] established a DC torque motor model based on the system generator platform and only designed a current loop control system based on PI controller. Liu et al. [14] contrived a new method of DC brushless motor speed control system based on radial basis function neural network control, and the simulation results demonstrated that it has excellent static and dynamic performance. However, these methods have poor disturbance rejection for motor angle control. Zhao et al. [15] proposed a quadruple closed-loop PID control strategy based on angle loop + angle acceleration loop + velocity loop + current loop according to the LATM mathematical model. There are more parameters to be adjusted and it is laborious for design although the simulation results achieved the desired goal. Chen et al. [16] derived an ideal model of the single-phase LATM with cylindrical Halbach magnetic array according to theories and proposed a robust output feedback control with a high-gain observer to manage a model’s uncertainty and the angle-related nonlinear current-torque relationship so that the defined smooth trajectory can be accurately tracked by angle sensing only. Experiments verified the effectiveness of the method. Çolak et al. [17] researched the LATM mathematical modeling and control methods in the Simulink simulation environment and verified the dual closed-loop control method of angle loop + current loop and the triple closed-loop control method of angle loop + angle velocity loop + current loop, respectively. The experimental results met the design criteria, but the mathematical models established in the above methods could hardly visualize an LATM’s features. Besides, the controller parameters were mainly tuned based on experience technology, and whether the LATM was loaded is still not considered. Zhou et al. [18] established a simulation model of an LATM for a marine diesel engine’s governor based on Simulink software through the identification method, and devised a triple closed-loop PID controller based on genetic algorithm. The experimental results proved the method’s effectiveness and disturbance rejection, but the genetic algorithm [19,20] is easy to confuse researchers by the local optimum, and the method’s real-time performance is not satisfactory. Besides, due to the computational complexity of genetic algorithm and the local prematurity of the optimal solution, it is more used in the parameter tuning of single loop PID controller and is not competent for the multi parameter tuning brought by multiple control loops [21].

Therefore, a Simscape-based LATM integrative physical modeling method is firstly proposed in this paper, targeting shortcomings of the current technology by taking 38LXJ01-Z LATMs as the research objects, which can intuitively demonstrate the actual operational characteristics of a motor. Secondly, a PID parameter self-tuning method based on a constriction factor particle swarm optimization (CPSO) algorithm was broached since it is difficult to tune due to a large number of multi-loop cascade PID controller parameters. A set of controller parameters that satisfy indicator requirements are obtained after a closed-loop PID controller composed of angle loop + angle velocity loop + current loop is rapidly and globally studied for the optimal parameters based on the target where the system’s response speed, overshoot and stability are ensured to be the best. The method is simpler, faster, and specifically better in load resistance compared with the previous empirical tuning method.

The structure of this paper is as follows. The LATM physical modeling is displayed in Section 2. CPSO-PID-based controller design is presented in Section 3. Results and discussion are demonstrated in Section 4, and conclusion is in Section 5.

## 2. Integrated Physical Modeling of Limited-Angle Torque Motor

### 2.1. Structure and Principle

**Figure 1:**
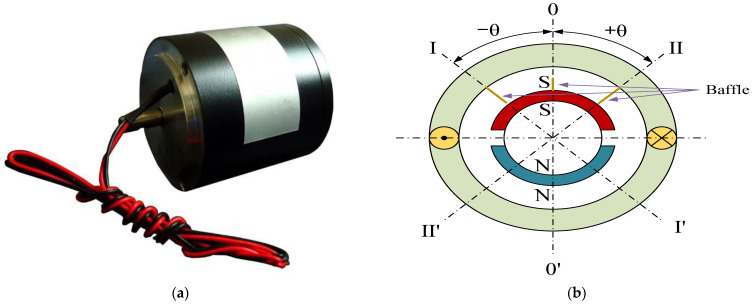
(**a**) 38LXJ01−Z limited-angle torque motor object; (**b**) Schematic diagram of motor structure section.

A 38LXJ01-Z LATM is deemed as the research object in this paper, which is specialized motors converting electrical signals into angular displacement at a certain angle with a certain torque output as presented in Figure 1. Such a LATM has no commutator and brush but has limited-angle physical module to confine the motor’s angle range. The motor stator magnetic slot number is 2, ±*θ* is the rotation angle range, 0-0′ is the motor reference/zero line and I-I’ and II-II’ are ±*θ*’s boundary lines where there is a baffle plate. The rotor will be blocked by the plate when reaching a boundary line, thus enabling the motor to rotate within the range of ±*θ*. The rotor is a two-pole structure consisting of permanent magnets. The stator and rotor magnetic fields of the motor are equally orthogonal, generating maximum electromagnetic torque with a demagnetizing or magnetizing potential within the operating range of ±*θ*. The motor will rotate in the reverse direction if a reverse current is applied to the stator coil; it will rotate in the forward direction if a forward current is applied to the stator coil [7].

### 2.2. Mathematical Model

A mathematical model specifically presented as follows is developed based on the working principle of LATMs [15]:

(1) Voltage balance equation

An LATM’s excitation system can be regarded as two excitation coils: one is a wound electric excitation coil, and the other is a permanent magnet excitation coil. The coils form circuits with the power supply, respectively, to obtain the voltage equation as:

(1) $$Ri_{1}+\frac{d\psi_{1}}{dt}=u_{1}$$

(2) $$\frac{d\psi_{2}}{dt}=u_{2}$$

where $u_{1}$ and $u_{2}$ are voltages of the two excitation coils respectively; $\psi_{1}$ and $\psi_{2}$ are flux linkages, and the resistance R is the equivalent resistance of the wound coil. The magnetic potential generated by the permanent magnet of magnetic steel is set to be $F_{2}$, and the number of turns of the permanent magnet coil is the same as that of electric excitation coils as *n*, so the current through the permanent magnet excitation coil is:

(3) $$i_{2}=\frac{F_{2}}{n}$$

Expressions of the flux linkages are:

(4) $$\psi_{1}=L_{11}i_{1}+L_{12}i_{2}$$

(5) $$\psi_{2}=L_{21}i_{1}+L_{22}i_{2}$$

where $L_{11}$ and $L_{22}$ are the self-inductance of coil 1 and coil 2, respectively. There is mutual inductance between the two coils, so $L_{12}=L_{21}$. The voltage balance equation is as follows:

(6) $$Ri_{1}+\frac{d}{dt}\left(L_{11}i_{1}+L_{12}i_{2}\right)=u_{1}$$

(7) $$\frac{d}{dt}\left(L_{21}i_{1}+L_{22}i_{2}\right)=u_{2}$$

The permanent magnet coil of magnetic steel can be deemed as a virtual electric coil, so this coil’s voltage equation can be ignored, but the flux it generates on the wound armature coil should be considered. Equations (6) and (7) can be simplified as follows when $L_{11}$ is a constant:

(8) $$R\cdot i+L\cdot \frac{di}{dt}+e=u$$

where $K_{e}=\frac{pN}{2\pi a}\cdot \phi$, ϕ=Bτl, and e is the counter-electromotive force (CEMF). $K_{e}\;$ is the motor’s CEMF coefficient, ϕ is the flux per pole, *N* is the total number of armature conductors, *a* is the number of branch pairs, *p* is the number of pole pairs, *B* is the average flux density per pole, *l* is the effective length of the conductors, and τ is the effective width of each pole on the armature core’s surface.

(2) Torque balance equation

The torque generated when the motor is working includes the electromagnetic torque output by the motor, the resistance torque produced by the motor’s mechanical damping, the load torque borne on the motor’s rotating shaft, and the resistance torque when the rotor reaches the angle boundary and collides with the motor’s physical baffle plate. The LATM’s torque balance equations are established according to the laws related to kinematics, as demonstrated in Equations (9)–(11):

(9) $$J\frac{d^{2}\theta }{dt^{2}}=M-D\frac{d\theta }{dt}-M_{l}$$

(10) $$J\frac{d^{2}\theta }{dt^{2}}=M-D\frac{d\theta }{dt}-M_{l}-M_{x1}$$

(11) $$J\frac{d^{2}\theta }{dt^{2}}=M-D\frac{d\theta }{dt}-M_{l}-M_{x2}$$

where J is the torque inertia, D is the viscous damping coefficient, M is the electromagnetic torque and $M=K_{T}\cdot i$, $M_{l}$ is the load torque, $K_{T}$ is the electromagnetic torque coefficient, $M_{x1}$ is the baffle plate’s instantaneous torque where $M_{x1}=\infty$ at the contact moment, and $M_{x2}$ the baffle plate’s steady-state torque whose expression is $M_{x2}=M-M_{l}$. The torque balance equation is as shown in Equation (9) when the LATM rotates; the torque balance equation in the transient state is as presented in Equation (10) when the motor reaches the baffle plate, i.e., the maximum angular displacement, and the motor immediately stops; the LATM’s torque balance equation is as demonstrated in Equation (11) when the motor reaches the baffle plate and is in the steady-state, and the reverse torque generated by the plate should be equal to the forward torque imposed by the motor’s rotor on the plate, i.e., the interaction torque. The output torque is zero at that time.

The voltage-related transfer function of the motor’s angle could be obtained as follows according to the voltage balance equation and the torque balance equations, when the LATM operates without load within the angle range:

(12) $$\frac{\theta \left(S\right)}{U\left(S\right)}=\frac{1}{\frac{JL}{K_{T}}S^{3}+\frac{RJ+LD}{K_{T}}S^{2}+\left(\frac{RD}{K_{T}}+K_{e}\right)S}$$

Based on $\omega =\frac{d\theta }{dt}$, Equation (9) among the torque balance equations can be transformed to:

(13) $$J\frac{d\omega }{dt}=M-D\omega -M_{l}$$

The voltage-related transfer function of the motor’s angle velocity could be solved combined with the voltage balance equation. The expression is presented as follows:

(14) $$\frac{W\left(S\right)}{U\left(S\right)}=\frac{1}{\frac{JL}{K_{T}}S^{2}+\frac{RJ+LD}{K_{T}}S+\left(\frac{RD}{K_{T}}+K_{e}\right)}$$

### 2.3. Integrated Physical Model

Building a physical model of the LATM is conducive to further demonstrating its real operating characteristics, so a Simscape-based [22] LATM integrative physical model is established in this paper, where motor components mainly include a motor angle measuring sensor, a motor driver, and loads.

#### 2.3.1. Motor

**Figure 2:**
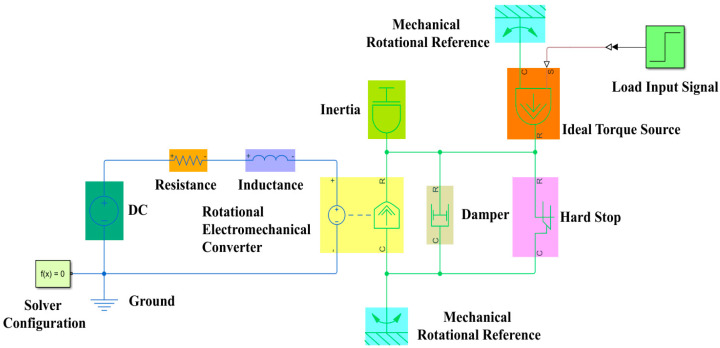
Physical model of limited-angle torque motor.

Firstly, the LATM-related parameters listed in Table 1 are accurately identified according to the established mathematical model and the measurement data. Secondly, a Simscape-based LATM ontology physical model is established as demonstrated in Figure 2 according to the parameters.

**Figure 3:**
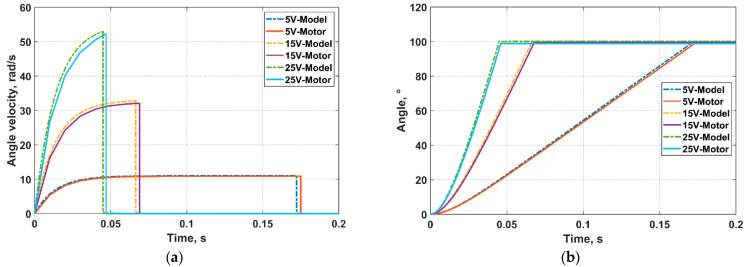
Comparison results between motor and physical model. (**a**) Angle velocity. (**b**) Angle.

In order to verify the accuracy and validity of the designed physical model, 5 V, 15 V, and 25 V forward driving voltages were input to the LATM and the Simscape-based motor model under the same conditions, respectively. The comparison results are displayed in Figure 3. In Figure 3a, the maximum rotational speed output by the motor model is 10.986 rad/s, and the rotational speed measured in the motor test is 10.890 rad/s when 5 V voltage is set as input, so the matching precision between the model and the motor is 99.13%, and their boundary reaching times are 0.172 s and 0.175 s, respectively; the maximum rotational speed output by the motor model is 32.788 rad/s, and the rotational speed measured in the motor test is 32.062 rad/s when 15 V voltage is set as input, so the matching precision between the model and the motor is 97.78%, and their boundary reaching times are 0.066 s and 0.069 s, respectively; the maximum rotational speed output by the motor model is 52.996 rad/s, and the rotational speed measured in the motor test is 52.273 rad/s when 25 V voltage is set as input, so the matching precision between the model and the motor is 98.64%, and their boundary reaching times are 0.045 s and 0.047 s, respectively. It is obvious that the model simulated rotational speed is slightly faster than the motor’s actual speed. Measured angles of the model and the motor corresponding to different input voltages are demonstrated in Figure 3b. The motor angles slightly deviated from 100° due to practical measurement errors. The Simscape-based physical model’s simulation accuracy is over 97% according to motor tests under typical operating voltages, which has proved the accuracy and validity of the Simscape-based model. Thus, the model can be adopted in designing and verifying control methods.

Meanwhile, as shown in Figure 3, the LATM is a non-linear object, and the response characteristics of the LATM do not change linearly when the driving voltage changes linearly at equal intervals of 5 V, 15 V, and 25 V. When the driving voltage is 5 V, the magnetic field is weak. This caused a small angle velocity to drive angle to the maximum value; when the driving voltage is 15 V, the magnetic field is strong. This caused a large angle velocity to drive angle to the maximum value; when the driving voltage is 25 V, the magnetic field is stronger. This caused a larger angle velocity to drive angle quickly to the maximum value. Therefore, this feature needs to be considered.

#### 2.3.2. Angle Measuring Sensor

Common motor angle and angle velocity measuring transducers mainly include Hall sensor, photoelectric sensor, and rotary variable differential transformer (RVDT). Fuel metering apparatus in aviation often uses an RVDT as its motor angle velocity and angle measuring transducer. Generally, an RVDT is connected coaxially with a motor, converts the motor’s position signals into electrical signals, and realizes higher measurement accuracy according to angle signals calculated by a solver. Therefore, an RVDT is selected as the angle measuring transducer in this paper.

**Figure 4:**
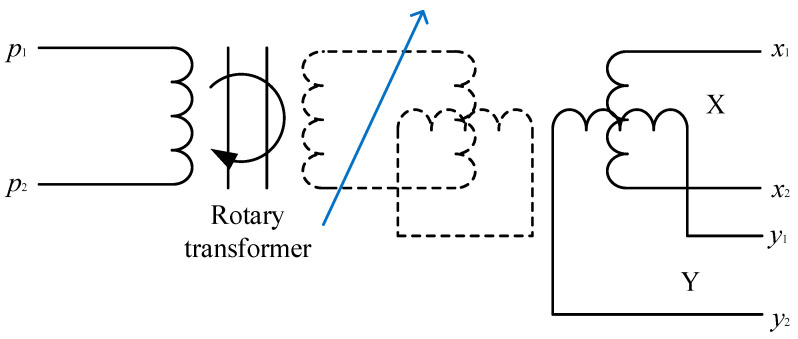
RVDT structure.

In principle, an RVDT is equivalent to a rotatable transformer and is similar with a two-stage two-phase wound induction motor in structure, whose stator and rotor are generally laminated with silicon steel or iron-nickel alloy sheets, and the angle between two secondary coil windings is 90°. Primary coil windings are AC excitation windings. As the rotor angle changes, the relative coupling between the primary and secondary windings also varies. Each of the two secondary coil windings produces corresponding induced electromotive force (EMF). The number of turns and the wire diameter of the stator windings of coils X and Y are the same in Figure 4. The two secondary windings X and Y will output induced voltages when excitation windings p1 and p2 are excited with a certain frequency of AC voltages, the amplitude of which is associated with the rotor’s rotational angle. A solver can obtain values of angle and angle velocity from electrical signals according to the output voltages at both ends of coils X and Y [23,24].

The voltage balance equation of coils and the induced EMF equation of secondary coils are as follows, according to the electromagnetism and Kirchhoff’s voltage law:

Kirchhoff’s voltage law:

(15) $$u+e+e_{\sigma }=K\cdot i$$

(16) $$u=K\cdot i+\left(-e_{\sigma }\right)+\left(-e\right)=K\cdot i+L_{\sigma }\frac{di}{dt}+\left(-e\right)$$

where u is the excitation voltage, e is the primary magnetic induced EMF, $e_{\sigma }$ is the flux leakage voltage, $L_{\sigma }$ is the coil inductance, K is the transformer ratio.

Transformer’s working principle:

(17) e≈u

(18) $$\frac{U_{1}}{U_{2}}\approx \frac{E_{1}}{E_{2}}=\frac{n_{1}}{n_{2}}=K$$

Equations of RVDT mathematical principles:

(19) $$v_{x}=K\cdot \cos(N_{p}\cdot \theta )\cdot v_{p}$$

(20) $$v_{y}=K\cdot \sin(N_{p}\cdot \theta )\cdot v_{p}$$

where $N_{p}$ is the number of an RVDT’s pole pairs, $v_{x}$ is the voltage at both ends of the secondary coil X, $v_{p}$ is the primary coil’s excitation voltage, $v_{y}$ is the voltage at the both ends of the secondary coil Y, and θ is the motor rotor’s rotational angle. The primary magnetic field was decomposed on the two secondary coils which formed an angle of 90°, and the induced EMF was generated on coils X and Y according to the transformer’s working principle. Electrical signals were transmitted to the solver unit through the voltage output ports of $x_{1}x_{2}$ and $y_{1}y_{2}$.

**Figure 5:**
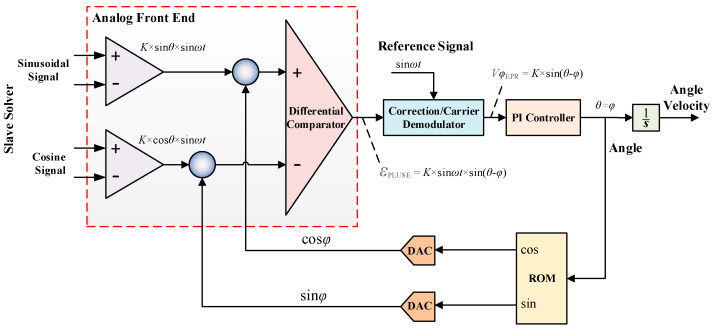
Basic principle of RVDT solution unit.

RVDT’s solver acquired the rotor’s current rotational angle (*θ*) by calculating the coupled voltage signals of the two stator coils. The principal block diagram of the PID loop-based angle solver adopted in this paper is presented in Figure 5, and the architecture can be divided into two parts, namely analog signals and digital signals. The analog front-end consists of a programmable gain amplifier and a comparator, and the analog front-end mainly realizes noise removal and provision of correct DC bias; the digital feedback loop is comprised of a filter and a PID controller. The digital angle information is firstly assumed and digitally processed by the sine and cosine table stored in the storage, then calculated by the sine and cosine digital-to-analog converter (DAC), and finally multiplied with the RVDT’s signals. The solving principle of the RVDT’s solver is to obtain the coupling signal (K⋅sinθ⋅sinωt, K⋅cosθ⋅sinωt) of the excitation signal (sinωt) on the stator winding, which is differentially compared, rectified and demodulated to obtain the PID controller’s error signal K⋅sin (θ−φ). At the end, errors are eliminated by PID regulation, thus obtaining the RVDT’s angle.

#### 2.3.3. Motor Driver

**Figure 6:**
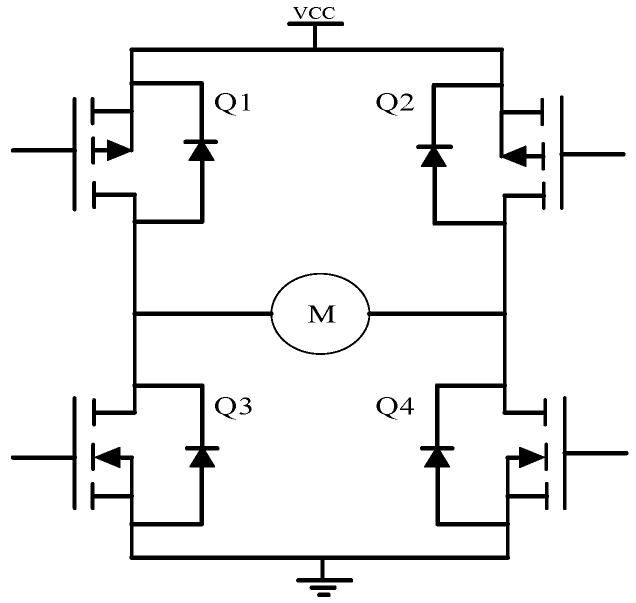
Schematic diagram of “H-bridge”.

The classical “H-Bridge” and “PWM (Pulse Width Modulation)” drive circuits are often employed to control the motor’s rotation direction and speed in the LATM control system [25]. The “H-Bridge” consists of four field-effect tubes, namely two PMOS (Positive channel Metal Oxide Semiconductor) in the upper arm and two NMOS (N-Metal-Oxide-Semiconductor) in the lower arm, and the control of motor’s rotation direction and speed is realized by controlling the sequence of the four MOS tubes to connect input signals and PWM values. The motor rotates clockwise when MOS tubes at Q1 and Q4 connect input signals as presented in Figure 6; it rotates anticlockwise when MOS tubes at Q2 and Q3 connect input signals; the motor’s speed can be controlled by changing the PWM signal’s duty cycle when the control signal is PWM signal [26].

#### 2.3.4. Load

A LATM directly controls the metering valve angle by levers and gears to meter fuel. The valve components and the effect of fuel pressure on the motor’s rotating shaft can be equivalently regarded as a certain load disturbance. A motor’s load model is often established with an ideal torque source module + mathematical signal source or mechanical spring module + gear set module in the motor’s integrative component models.

**Figure 7:**
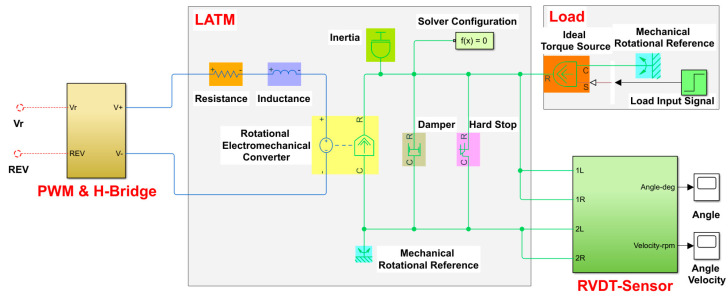
Integrated physical simulation model of limited-angle torque motor.

Therefore, the LATM integrative physical simulation model of fuel metering apparatus established according to the method is demonstrated in Figure 7.

## 3. CPSO-PID Controller Design

The LATMs are characterized by high rotational inertia ratio, fast dynamic response, outstanding linearity, compact structure, low steady-state power, and bidirectional controllability. However, the position control system composed of an LATM has severe nonlinearity, and it is arduous for the traditional series correction, speed feedback, and compound control methods to achieve the ideal control effect. Therefore, a triple closed-loop cascade PID controller (angle loop + angle velocity loop + current loop) is established in this paper to achieve high-precision position control of an LATM. The more cascade PID controllers are, the more parameters are to be adjusted. Poor effect and huge workloads will be seen if parameters are adjusted by totally relying on professional experience. Therefore, a CPSO-based PID parameter quick self-tuning method is proposed in this paper. The PID parameters are automatically adjusted by the CPSO algorithm, which not only greatly reduces the workloads, but also obtains better control effect.

### 3.1. CPSO Algorithm

The particle swarm optimization algorithm is a simple and effective swarm intelligence optimization algorithm. The traditional particle swarm algorithm depends largely on its parameters, and improper setting of parameters is easy to confuse researchers by local optimal solutions with poor results [27], while CPSO algorithm can address this problem [28]. Details are as follows:

The fitness value corresponding to each particle’s position ***x****_i_* can be calculated according to the objective function if the *i*th particle of a swarm *x* = (*x*_1_, *x*_2_, …, *x_n_*) consisting of *n* particles in a D-dimensional search space is represented as a D-dimensional vector *x_i_* = (*x_i_*_1_, *x_i_*_2_, *x_i_*_3_,…, *x_i_*_D_)^T^, which demonstrates not only the *i*th particle’s position in the search space but also a potential optimal solution to the original problem. The *i*th particle’s velocity can be denoted as *v_i_* = (*v*_1_, *v*_2_, …, *v_D_*)^T^, whose individual extremum, i.e., the best position experienced by the *i*th particle in the D-dimensional space, has the best fitness at *pb_i_* = (*p_i_*_1_, *p_i_*_2_, …, *p_iD_*). The swarm’s global extremum, i.e., the best position experienced by all its particles, can be presented as *pg* = (*pg*_1_, *pg*_2_, …, *pg_D_*)^T^.

The mathematical model for each particle’s movement and position update in the CPSO algorithm is shown as follows:

(21) $$v_{i}\left(k+1\right)=\chi \left[v_{i}\left(k\right)+r_{1}c_{1}\left(pb_{i}\left(k\right)-p_{i}\left(k\right)\right)+r_{2}c_{2}\left(pg\left(k\right)-p_{i}\left(k\right)\right)\right]$$

(22) $$p_{i}\left(k+1\right)=p_{i}\left(k\right)+v_{i}\left(k+1\right)$$

where *i* is the particle index; *k* is the number of the algorithm’s iterations; $p_{i}\left(k\right)$ and $v_{i}\left(k\right)$ are the *i*th particle’s position and velocity in the search space at the *k*th iteration, respectively; *r*_1_ and *r*_2_ are random numbers between zero and one. *c*_1_ and *c*_2_ are acceleration factors, the sum of which is greater than 4, i.e., *C* = *c*_1_ + *c*_2_ > 4; χ is the compressibility factor, which is calculated as follows:

(23) $$\chi =\frac{2}{\left|2-C-\sqrt{C^{2}-4C}\right|}$$

It decreases each particle’s velocity as the number of iterations increases.

$pb_{i}$ and pg are updated as follows during each iteration.

(24) $$pb_{i}\left(k+1\right)=\{\begin{array}{ll} pb_{i}\left(k\right) & \;\mathrm{if}\;J\left(p_{i}\left(k+1\right)\right)\geq J\left(pb_{i}\left(k\right)\right)\\ p_{i}\left(k+1\right) & \;\mathrm{if}\;J\left(p_{i}\left(k+1\right)\right)<J\left(pb_{i}\left(k\right)\right) \end{array}$$

(25) $$pg\left(k+1\right)=\{\begin{array}{ll} pg\left(k\right) & \mathrm{if}\;J\left(pb_{i}\left(k+1\right)\right)\geq J\left(pg\left(k\right)\right)\\ pb_{i}\left(k+1\right) & \mathrm{if}\;J\left(pb_{i}\left(k+1\right)\right)<J\left(pg\left(k\right)\right) \end{array}$$

where $J\left(p_{i}\left(k+1\right)\right)$ is CPSO’s fitness function.

**Figure 8:**
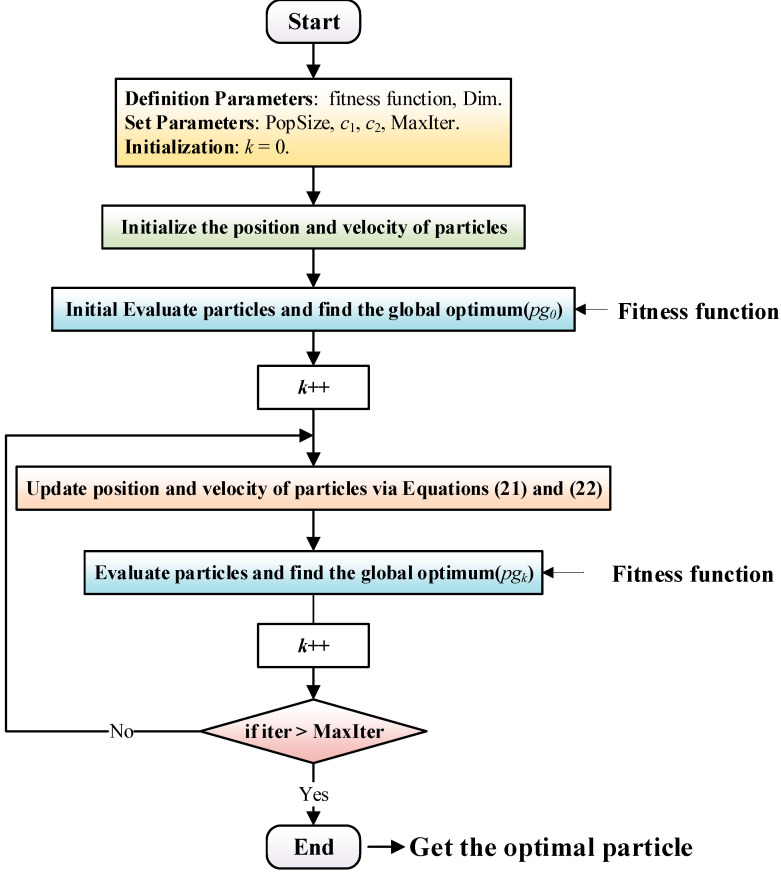
Principle of CPSO algorithm.

Therefore, a CPSO algorithm concrete realization flow is displayed in Figure 8. Firstly, it requires the following parameters such as fitness function, particle dimension (Dim), population size (PopSize), the maximum iteration number (MaxIter), *c*_1_, *c*_2_, and *k*. Then, it is essential to initialize the position and velocity of particles as well as evaluate particles and find the global optimum (*pg_0_*). Next, it will enter the loop iteration process and the position and velocity of particles via Equations (21) and (22) is updated. Subsequently, these particles need to be evaluated and find the global optimum (*pg_k_*) again by comparing it with the previous global optimal value. Finally, the optimal particle is obtained when the termination condition (iter > MatIter) is satisfied.

### 3.2. Design of Multi-Loop Cascade PID Controller

**Figure 9:**
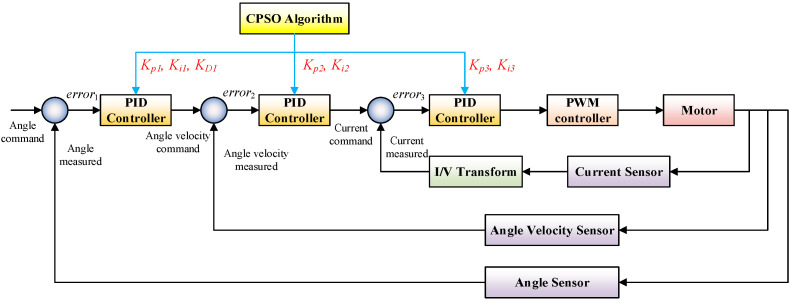
Triple closed loop cascade PID control of LATM based on CPSO algorithm.

In the design of a triple closed-loop controller, from outside to inside are the angle loop, the angle velocity loop, and the current loop. The angle velocity loop can play a role in suppressing the fluctuation of the angle position, improving the steady-state accuracy, response speed, and enhancing disturbance rejection. In addition, the angle velocity loop also uses a PI controller, and the system will be instable if the differential link D is added. The triple closed-loop control strategy of the angle loop + angle velocity loop + current loop adopted by the LATM is presented in Figure 9.

In this paper, the CPSO algorithm is adopted for PID controller parameter self-tuning. The more optimization variables exist, the more efforts are paid in computation, and the longer time is consumed. Thus, the angle loop uses a PID controller with the proportional, the integral and the differential coefficients of *K_p_*_1_, *K_i_*_1_, and *K_d_*_1_. The angle velocity loop employs a PI controller with the proportional and the integral coefficients of *K_p_*_2_ and *K_i_*_2_. The current loop adopts a PI controller with the proportional and the integral coefficients of *K_p_*_3_ and *K_i_*_3_. Therefore, the optimization variable is selected as:

(26) $$x=\left[\begin{array}{ccccccc} K_{p1} & K_{i1} & K_{d1} & K_{p2} & K_{i2} & K_{p3} & K_{i3} \end{array}\right]$$

The integral of time and absolute error (ITAE) is adopted in this paper as the performance index to establish an optimization seeking objective function in order to ensure the response speed, overshoot, and stability of the system, whose expression is:

(27) $$J_{ITAE}=\int_{0}^{\infty }t\left|e\left(t\right)\right|dt$$

where $J_{ITAE}$ is the fitness evaluation function of the CPSO algorithm.

Finally, a CPSO-PID controller design method is obtained according to the LATM optimal control method proposed in this paper, as shown in Algorithm 1. It mainly includes two parts: Offline Parameters Self-tuning and Online PID Control.
**Algorithm 1:** CPSO-PID Controller Design
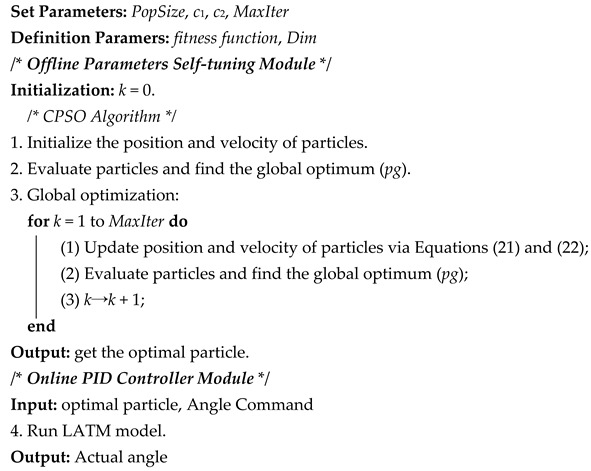



## 4. Results and Discussion

**Figure 10:**
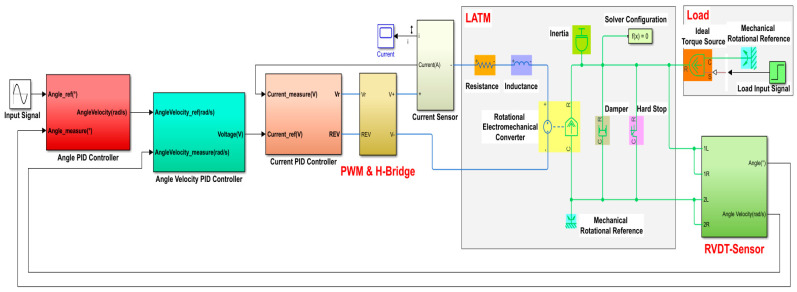
Simulation model of limited-angle torque motor control system based on Simscape.

A Simscape-based LATM multi-loop cascade PID control system is established in this paper, as demonstrated in Figure 10, mainly including a cascade PID controller and a motor’s integrative physical model consisting of the LATM, load, a RVDT sensor, a driver, and other modules.

**Figure 11:**
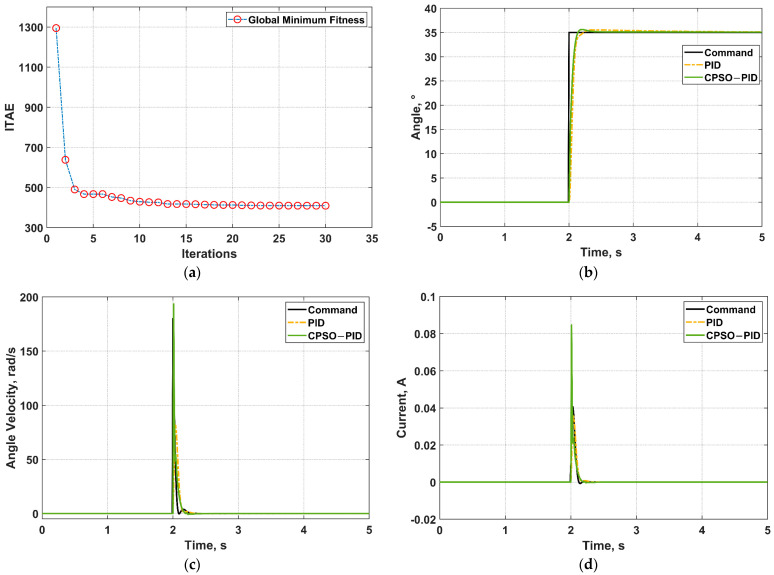
Comparison results of CPSO−PID and PID in the case of angle step signal. (**a**) Global minimum fitness convergence process; (**b**) Angle control; (**c**) Angle velocity control; (**d**) Current control.

The Simscape-based motor model is deemed as the object in this paper to tune PID parameters according to the CPSO algorithm’s principle. CPSO related setting’s parameters are the number of particle swarms is: PopSize = 30, Dim = 7, MaxIter = 30, *c*_1_ = 3.5, and *c*_2_ = 2. The optimization seeking results are presented in Figure 11. The CPSO’s ITAE convergence is demonstrated in Figure 11a, where it is obvious that the asymptotic convergence remains constant after reaching a certain value within the set number of iterations. The corresponding global optimal particle finally obtained from optimization will be applied to the motor control as the cascade PID parameter. The global optimal particle is:

(28) $$x=\left[\begin{array}{ccccccc} 4.88016 & 1.20065 & 0.03159 & 0.001337 & 0.01889 & 63.77460 & 4091.44024 \end{array}\right]$$

As in Figure 11b,c, CPSO−PID’s performance index is better than PID’s. The CPSO−PID can respond more quickly with more accurate steady-state control (as shown in Table 2). The control performance increases by 40% by virtue of PID parameters tuned by the CPSO algorithm other than those tuned by professional experience. Moreover, the calculation is simpler.

**Figure 12:**
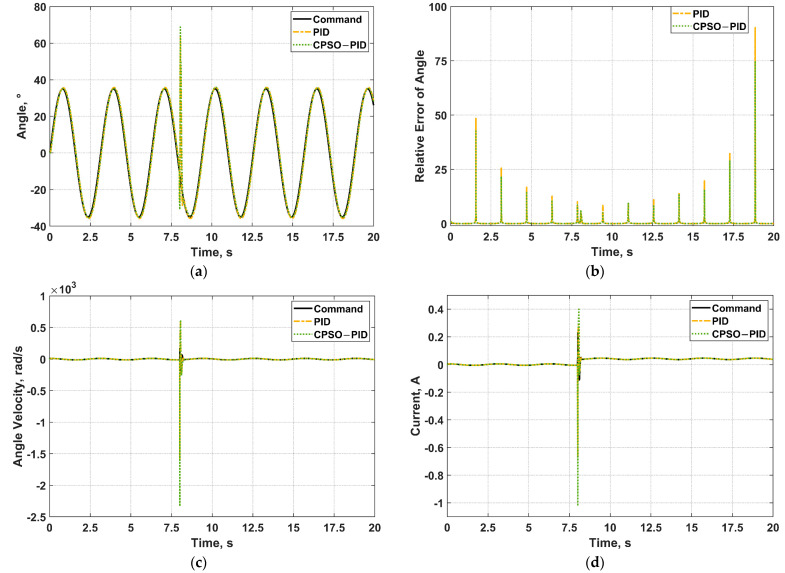
The simulation result when the angle command input signal is a sine wave with frequency of 2 rad/s adding a step load at *t* = 8 s. (**a**) Angle control; (**b**) Relative error of angle; (**c**) Angle velocity control; (**d**) Current control.

The control effect of CPSO−PID is compared with that of PID as shown in Figure 12 if the angle command input is a standard sine wave signal with a given frequency of 2 rad/s and an amplitude of 35°. From the angle relative error graph (Figure 12b), it is conspicuous that the control accuracy of CPSO−PID is higher. A step load of 0.5 N·cm is added at the 8th second and continuously acts. The motor’s angle, angle velocity, and current change abruptly as presented in Figure 12a,c,d, but recover quickly and maintain favorable control effect.

**Figure 13:**
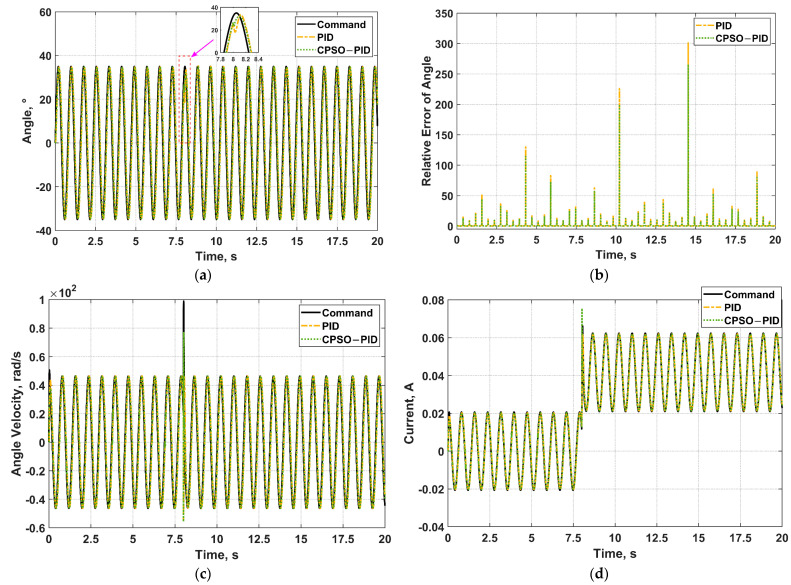
The simulation result when the angle command input signal is a sine wave with frequency of 8 rad/s adding a step load at *t* = 8 s. (**a**) Angle control; (**b**) Relative error of angle; (**c**) Angle velocity control; (**d**) Current control.

To verify the effectiveness and robustness of CPSO−PID controller, a sine wave with a frequency of 8 rad/s was set as an input signal of angle command. The simulation result is illustrated in Figure 13. Figure 13a shows that control effect of the CPSO−PID is superior to the PID. When adding a step load at *t* = 8 s, the CPSO−PID has a stronger anti-disturbance ability. Figure 13a displays that the CPSO−PID has better controller accuracy than PID. Figure 13c,d demonstrated that the angle velocity and current loop are also well controlled. Therefore, The CPSO−PID controller designed in this paper has good robustness.

**Figure 14:**
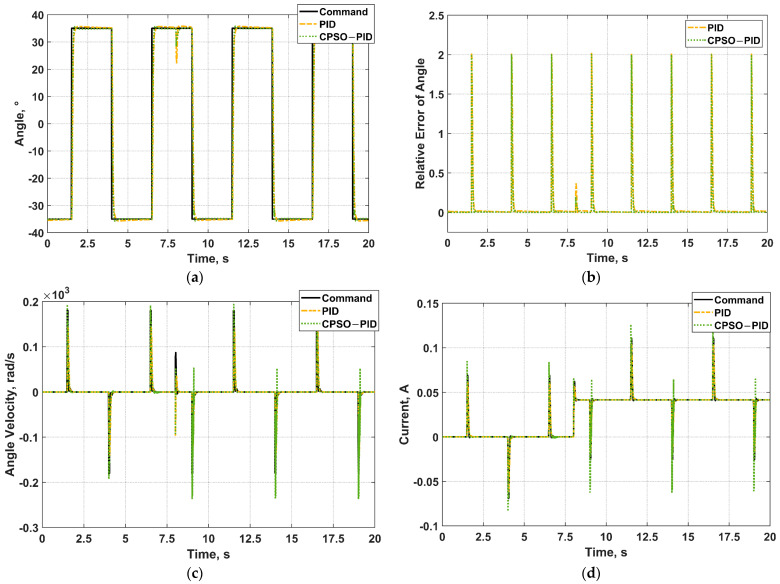
The simulation result when the angle command input signal is a square wave with frequency of 0.2 Hz adding a step load at *t* = 8 s. (**a**) Angle control; (**b**) Relative error of angle; (**c**) Angle velocity control; (**d**) Current control.

The control effect of CPSO−PID is compared with that of PID, as shown in Figure 14, if the angle command input is a standard square wave signal with a given frequency of 0.2 Hz and an amplitude of 35°. From the angle relative error graph (Figure 14b), it is conspicuous that the control accuracy of CPSO−PID is higher. A step load of 0.5 N·cm is added at the 8th second and continuously acts. The motor’s position, angle velocity, and current change abruptly as presented in Figure 14a,c,d, but recover quickly and maintain favorable control effect.

Therefore, the PID control performance increases by 40% by virtue of parameters automatically tuned by the CPSO algorithm other than those tuned by professional experience according to the comparative simulation results, having better dynamic and steady-state performance. Moreover, the design method is simpler and consumes less time.

**Figure 15:**
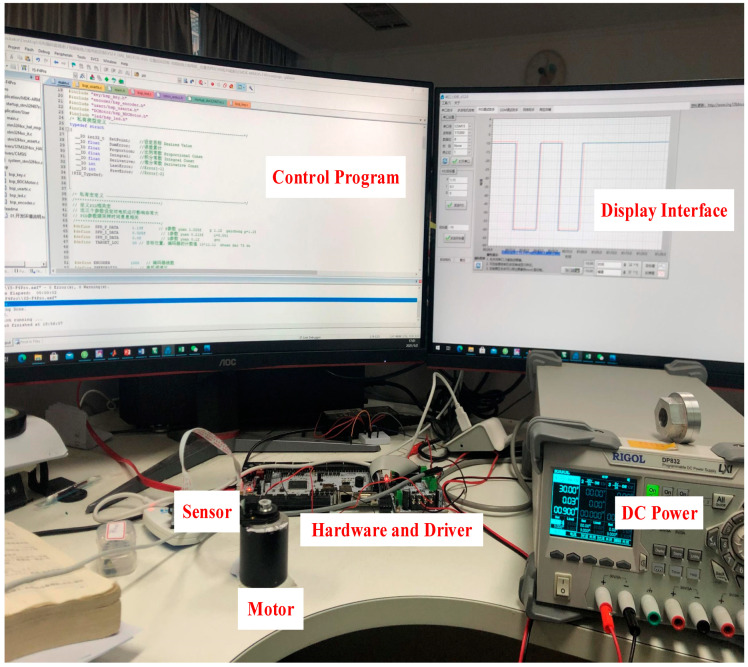
LATM control experiment environment.

Finally, a hardware experimental test environment is also built, as presented in Figure 15 in this paper, to further verify the effectiveness of the designed optimal controller, which mainly includes a motor, sensor, hardware and driver, control programs, the display interface, DC power, and other devices. The experimental results are consistent with the simulation results, and the time to regulate the motor is short. There is almost no overshoot, and the steady-state error is less than 0.2%. The optimal control is proved effective, further guiding the engineering design.

## 5. Conclusions

The Simscape-based fuel metering apparatus’ LATM integrative physical modeling method was studied in this paper, which was compared with a real motor for verification. The modeling accuracy reaches 97%, which can intuitively demonstrate the motor’s operating characteristics. In the end, a CPSO-based PID parameter self-tuning method was proposed in this paper to study globally for the optimal parameters since parameters of the triple closed-loop cascade PID controller can hardly be tuned via experience. According to the comparative simulation and experiment results, the system control performance increases by 40% because of parameters tuned by the proposed method other than those tuned by professional experience. The control system has better disturbance rejection when sine wave and square wave signals were used as input commands of angle, respectively. Besides, the method put forward in this paper is smarter, simpler, and faster during the entire design.

## Figures and Tables

**Table 1:** Parameters of 38LXJ01-Z limited-angle torque motor.

Parameters	*R* (Ω)	*L* (H)	*K_e_* (V/(rad/s))	*J* (kg·m^2^)	*K_T_* (N·m/A)	*D* (N·m(rad/s))	Angle Range (°)
Value	81.15	1.5	0.12	2 × 10^−8^	0.12	5 × 10^−4^	±100

**Table 2:** Comparison of CPSO-PID and PID performance indexes.

Performance Index	PID	CPSO−PID
Rise time	0.2281 s	0.1386 s
Overshoot	1.5%	1.5%
Setting time	1.1 s	0.3 s
Steady-state accuracy	0.29%	0.19%

## References

[1] Yao H. (2014). Full Authority Digital Electronic Control System for Aero-Engine.

[2] Shang Y., Guo Y.Q., Wang J.C., Wang L. (2013). Modeling and Performance Analysis of Augmented-fuel Metering Unit for Turbofan Engine. Aeroengine.

[3] Krishna P.M., Kannan N. (1996). Brushless DC limited angle torque motor. Proceedings of the International Conference on Power Electronics, Drives and Energy Systems for Industrial Growth.

[4] Liu J., Zhao Y., Fan X., Zhang X. (2019). The Invention Relates to a Stator Double Excitation Torque Motor with Limited Turning Angle. Micromotors.

[5] Guo L., Zheng C.L., Wang H. (2021). Design and Simulation of Semi—Immersed Limited Angle Torque Motor. Small Spec. Electr. Mach..

[6] Tsai C.C., Lin S.C., Huang H.C., Cheng Y.M. (2009). Design and control of a brushless DC limited-angle torque motor with its application to fuel control of small-scale gas turbine engines. Mechatronics.

[7] Nasiri-Zarandi R., Mirsalim M., Cavagnino A. (2015). Analysis, optimization, and prototyping of a brushless DC limited-angle torque-motor with segmented rotor pole tip structure. IEEE Trans. Ind. Electron..

[8] Wu S., Zhao X., Jiao Z., Luk P.C.K., Jiu C. (2016). Multi-objective optimal design of a toroidally wound radial-flux Halbach permanent magnet array limited angle torque motor. IEEE Trans. Ind. Electron..

[9] Hekmati P., Yazdanpanah R., Mirsalim M., Ghaemi E. (2016). Radial-flux permanent-magnet limited-angle torque motors. IEEE Trans. Ind. Electron..

[10] Li Y., Ma P., Wang Q., Zhao M. (2020). Analysis, Modeling, and Verification of Limited Angle Torque Motors with Irregular Slot Numbers for Performance Improvement. IEEE Trans. Energy Convers..

[11] Zhang Y., Smith I.R., Kettleborough J.G. (1999). Accurate tracking control of a limited angle torque motor. Electr. Mach. Power Syst..

[12] Xiao R., Zhou M., Hao S.G. (2008). Design of Position Servo Driver for DC Limited Angle Torque Motor. Micromotors.

[13] Li B.R., Li X.F., Yan C.X., Yu P. (2013). Simulation of DC Torque Motor Model and Control System Based on System Generator. Instrum. Tech. Sens..

[14] Liu H., Wang Z., Yang F. (2013). A limited Angle Torque Motor Desig. Micromotors.

[15] Zhao H.W. (2008). Design and Research on LABLTM Used for the Electronic Governor’s Actuator of Diesel Engine. Master’s Thesis.

[16] Chen S.L., Kamaldin N., Teo T.J., Liang W., Teo C.S., Yang G., Tan K.K. (2015). Toward comprehensive modeling and large-angle tracking control of a limited-angle torque actuator with cylindrical Halbach. IEEE/ASME Trans. Mechatron..

[17] Çolak İ., Sahin M., Çakıroğlu S., Esen Z. (2016). Controller design for a limited angle torque motor and dsPIC implementation. Proceedings of the 2015 International Aegean Conference on Electrical Machines & Power Electronics (ACEMP), 2015 International Conference on Optimization of Electrical & Electronic Equipment (OPTIM) & 2015 International Symposium on Advanced Electromechanical Motion Systems (ELECTROMOTION).

[18] Zhou L., Tang Q., Tan H., Zhou T., Wang X., Wang Y., Chen H. (2021). Optimal Control of Limited Angle Torque Motor for Governor of Marine Diesel Engine. Mar. Electric..

[19] Malhotra R., Singh N., Singh Y. (2011). Genetic algorithms: Concepts, design for optimization of process controllers. Comput. Inf. Sci..

[20] Makarem S., Delibas B., Koc B. (2021). Data-driven tuning of PID controlled piezoelectric ultrasonic motor. Actuators.

[21] Zahir A.A.M., Alhady S.S.N., Othman W.A.F.W., Ahmad M.F. (2018). Genetic algorithm optimization of PID controller for brushed DC motor. Intelligent Manufacturing Mechatronics.

[22] Szántó A., Hajdu S., Deák K. (2022). Longitudinal Dynamic Modeling and Driving Cycle Tracking Control of an Electric-Driven Vehicle by Means of MATLAB/Simulink/Simscape. Period. Polytech. Transp. Eng..

[23] Sarma S., Agrawal V.K., Udupa S., Parameswaran K. (2008). Instantaneous angular position and speed measurement using a DSP based resolver-to-digital converter. Measurement.

[24] Ai L., Yang H. (2014). Shaft Angle Position Signal Detection Using the Rotary Transformer. Electron. Sci. Tech..

[25] Noorsal E., Rongi A., Ibrahim I.R., Darus R., Kho D., Setumin S. (2022). Design of FPGA-Based SHE and SPWM Digital Switching Controllers for 21-Level Cascaded H-Bridge Multilevel Inverter Model. Micromachines.

[26] Zhang H.Y., Zhang C. (2014). Theory and Control Method Study of Cascaded H-bridge Rectifier. Power Electron..

[27] Tran H.K., Chiou J.S. (2016). PSO-based algorithm applied to quadcopter micro air vehicle controller design. Micromachines.

[28] Pervez I., Sarwar A., Pervez A., Tariq M., Zaid M. (2021). An improved maximum power point tracking (MPPT) of a partially shaded solar PV system using PSO with constriction factor (PSO-CF). Advances in Electromechanical Technologies.

